# Analysis and Experiments of Resonant Coupling Wireless Power Transfer System for Nonuniform Powering of Multiple Sensors

**DOI:** 10.3390/s25072342

**Published:** 2025-04-07

**Authors:** Thuc Phi Duong, Ngoc Hung Phi, Bilal Ahmad, Sasani Jayasekara, Jong-Wook Lee

**Affiliations:** 1 Department of Electronics Engineering, Information and Communication System-on-Chip (SoC) Research Center, Kyung Hee University, 1732 Deogyeong-daero, Giheung, Yongin 17104, Gyeonggi-do, Republic of Korea; thuc@khu.ac.kr; 2 Department of Electronics and Information Convergence Engineering, Kyung Hee University, Yongin 17104, Gyeonggi-do, Republic of Korea; phingochung@khu.ac.kr (N.H.P.); bilal442@khu.ac.kr (B.A.); jaye130@khu.ac.kr (S.J.)

**Keywords:** wireless power transfer, multiple receivers, power distribution, resonance coupling

## Abstract

With a quickly increasing number of Internet of Things (IoT) involving different power levels, wireless power transfer (WPT) systems need the capability to deliver energy to multiple receivers simultaneously. The nonuniform powering of multiple receivers is also necessary, considering the different power levels that IoT sensors demand. This paper investigates asymmetric resonant coupling WPT systems for powering multiple receivers. We propose a simple method for achieving the specified power ratio of the multiple receivers using the equivalent circuit model and reflected impedance technique. The results are generalized for a system with an *N* number of multiple receivers. Experiments are performed for powering two receivers with power ratios of 1.5 and 2.5, which achieve a power transfer efficiency of 91.7% and 88.6%, respectively. Another experiment performed for powering four receivers, which have power ratios of 1.0, 1.5, 2.0, and 0.75, shows an efficiency of up to 89.9%, which agrees well with the simulation result. Our result shows that the distance between the source loop and the transmitting resonator can be varied to maximize efficiency without altering the power division.

## 1. Introduction

The rapid development of wireless power transfer (WPT) technology has shown good potential for charging the Internet of Things (IoT) without wiring constraints [1,2]. The potential is attributed to the new and efficient design of the resonator coupling WPT system. Several techniques have been proposed to overcome the limitations of conventional WPT [2,3,4,5,6,7,8,9,10,11,12,13,14,15,16,17,18,19,20,21,22,23,24,25,26,27,28,29,30,31,32]. In [3], strongly coupled resonance is utilized to extend WPT to a mid-range distance (~2 m). In [4], the variable coupling method is proposed to increase efficiency at an extended distance. In [5], frequency tuning is utilized to optimize the transmission efficiency when WPT is performed in an over-coupled region. In [6], the automatic impedance matching technique is realized to improve efficiency. Operating ranges of WPT have been extended using an intermediate coil [7] and an array of resonators [8]. In [9], a metamaterial inserted between a transmitter and a receiver is used to localize power to the desired location.

Additionally, multiple transmitters are investigated to improve the misalignment tolerance [10,11], and coupling-dependent output power stability [12,13]. Most of these works, however, provide techniques for the wireless powering of a single receiver [10,11,12,13,14,15,16,17,18,19,20]. With a quickly increasing number of IoT sensors and mobile devices demanding different power levels, a WPT system for the simultaneous charging and power control of multiple receivers is required. Various WPT methods for powering multiple receivers have been investigated [21,22,23,24,25,26,27,28,29,30,31,32].

In [21], an inductive coupling WPT system of multiple transmitters and receivers is investigated theoretically and experimentally. The result shows that multiple receivers make the system less sensitive to load change, and multiple transmitters increase overall efficiency. In [22], the frequency change by the coupling between multiple transmitters/receivers is investigated. In [23], a metamaterial is used between a transmitter and multiple receivers. In [24], an impedance inverter at the receiver side is proposed for impedance matching and power division. However, the analysis is limited to a relatively simple WPT system with two coils [22,23,24].

In [25], a mid-range WPT system with a single transmitter powering several receivers is analyzed. The result shows that the overall efficiency, when the system is appropriately tuned, is significantly greater than that of a system with a single receiver. In [26], the effect of cross-coupling between nonadjacent coils in multiple receivers is considered. Analysis and experiments are performed for powering multiple receivers using parity–time symmetry [27,28,29]. In [32], the frequency splitting in the resonant coupling WPT system, which consists of a large source coil and two small receivers, is investigated. Controlling the power delivered to multiple receivers is an important issue; however, in the works mentioned above, the power distribution for multiple receivers using resonant coupling has not been studied in detail.

This paper investigates an asymmetric resonant coupling WPT system for the nonuniform powering of multiple receivers. Using the equivalent circuit model and reflected impedance technique, we investigate a simple method for achieving the specified power ratio of the multiple receivers. We also derive the conditions for maximum efficiency for the specified power division ratio. The results are generalized for the WPT system with *N* receivers. Experiments are performed for powering two receivers with power ratios of 1.5 and 2.5, which achieve a power transfer efficiency of 91.7% and 88.6%, respectively. Another experiment performed for powering four receivers, which have different power ratios of 1.0, 1.5, 2.0, and 0.75, shows an efficiency of up to 89.9%. The results agree well with the simulated data. The contributions of this work can be summarized as follows:

(1) Nonuniform powering of multiple receivers: considering the different power levels that the IoT sensors demand, we present a simple method for achieving the specified power ratio of the multiple receivers using the equivalent circuit model and reflected impedance technique.
(2) Generalized multi-receiver approach: unlike prior studies focusing on single receivers or requiring complex impedance matching, our approach provides a simple approach of extending the analysis to multiple receivers and achieves relatively high efficiencies.
(3) Experimental validation of a non-invasive tuning method: using a large transmitter and multiple smaller receivers in an asymmetric WPT configuration, our experiments confirm that the desired power ratio can be achieved by adjusting receiver positions, eliminating the need for circuit modifications.

The proposed method can be applied to various WPT systems where a transmitter delivers power to multiple small receivers.

## 2. Analysis

### 2.1. Proposed WPT System for Multiple Receivers

**Figure 1:**
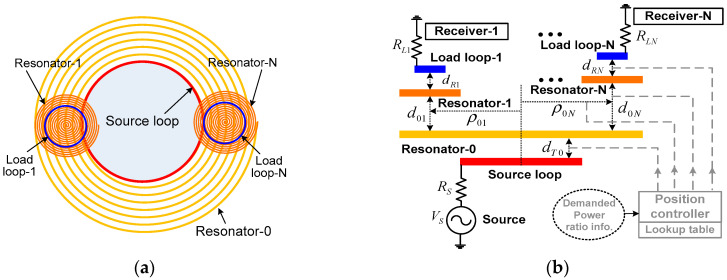
Schematic of the WPT system for powering multiple receivers: (**a**) top view, (**b**) side view.

Figure 1 shows a schematic of the proposed asymmetric WPT system with multiple receivers. A transmitter (receiver) consists of a pair of coils, which is a source (load) loop and a resonator. For high-quality factors, transmitting resonator-0 has multiple turns and uses self-capacitance for resonance. The size of the receiving resonator-*i* (*i* = 1 − *N*) is relatively smaller than the transmitting resonator-0, and lumped capacitors are used for resonance. The source loop is connected to a power source $V_{S}$ with resistance $R_{S}$, and it is in coaxial alignment with transmitting resonator-0 at a distance $d_{T0}$. The load loop-*i* connected to the load $R_{Li}$ has a distance $d_{Ri}$ from the receiving resonator-*i*. Between the resonator-*i* and resonator-0, there is a vertical distance of $d_{0i}$ and lateral displacement of $\rho_{0i}$ from the center of the resonator-0. This prototype uses power sensors to achieve the demanded power ratio. The system can add a position controller and a lookup table (not implemented in this prototype) for automated control. The lookup table can store the values of parameter sets ($d_{0i}$, $\rho_{0i},\;d_{Ri}$, and $d_{T0}$) corresponding to the measured power value. When the information for the desired power ratio is input, the controller can move the coils to the desired locations.

**Figure 2:**
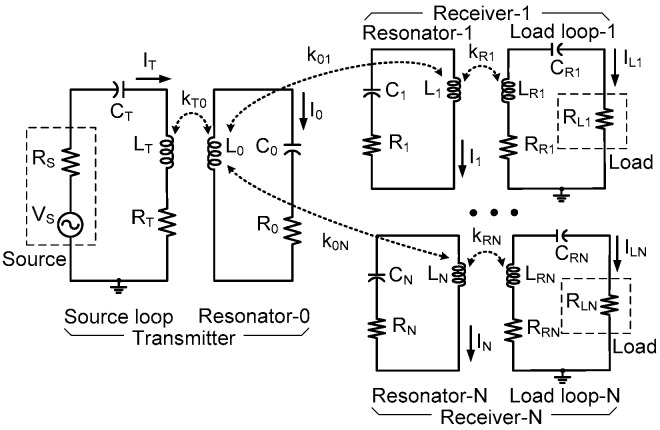
Equivalent circuit of the resonator-coupled WPT system with one transmitter and *N* receivers.

Figure 2 shows the equivalent circuit model of the WPT system with *N* receivers. The source and load loop-*i* are represented by lumped-circuit elements, $L_{T}$, $C_{T}$, $R_{T}$, and $L_{Ri}$, $C_{Ri}$, $R_{Ri}$, respectively (*i* = 1 − *N*). The transmitting resonator-0 coupled to the source loop is represented by $L_{0}$, $C_{0}$, and $R_{0}$. The receiving resonator-*i* is modeled using $L_{i}$, $C_{i}$, and $R_{i}$. Three coupling coefficients are defined: $k_{T0}$ between the source loop and resonator-0, $k_{0i}$ between resonator-0 and resonator-*i*, and $k_{Ri}$ between the load loop and resonator-*i*. In this work, the transmitter is much larger than the receivers.

We assume that the receiver locations do not overlap. Furthermore, we consider the case when the receivers are not clustered together. Under these conditions, the parasitic mutual coupling between nonadjacent receivers is much smaller than the primary coupling between transmitting resonator-0 and each receiver. We may also neglect the mutual coupling between adjacent receivers. This is the regime of interest of this paper. In practical applications, we can consider shielding each receiver to increase the isolation.

For analysis, all coils and resonators are assumed to oscillate at the same frequency ($f_{0}$ = 6.5 MHz). To simplify the notation, we define the load ($U_{Ri}$), transmitter ($U_{T0}$), and receiver ($U_{0i}$) coupling factors, which depend on normalized coupling coefficients (κ) and the loss rate (Γ), as

(1) $$U_{Ri}=\frac{\kappa_{Ri}^{2}}{\Gamma_{i}\Gamma_{Ri}},\;\;\;\;\;\;\;U_{T0}=\frac{\kappa_{T0}^{2}}{\Gamma_{T}\Gamma_{0}},\;\;\;\;\;\;\;U_{0i}=\frac{\kappa_{0i}^{2}}{\Gamma_{0}\Gamma_{i}},$$

which are the squares of the figure of merit, denoted by

(2) $$\frac{\kappa_{Ri}}{\sqrt{\Gamma_{i}\Gamma_{Ri}}},\;\;\;\;\;\;\;\frac{\kappa_{T0}}{\sqrt{\Gamma_{T}\Gamma_{0}}},\;\;\;\;\;\;\;\frac{\kappa_{0i}}{\sqrt{\Gamma_{0}\Gamma_{i}}}.$$

The normalized coupling coefficients are defined using

(3) $$\kappa_{\mathrm{Ri}}=\omega_{0}k_{\mathrm{Ri}}/2,\;\;\;\;\;\;\;\kappa_{T0}=\omega_{0}k_{T0}/2,\;\;\;\;\;\;\;\kappa_{0i}=\omega_{0}k_{0i}/2.$$

Here, the loss rates are defined as $\Gamma_{i}=\omega_{0}/2Q_{i}$, $\Gamma_{Ri}=\omega_{0}/2Q_{Ri}$, $\Gamma_{T}=\omega_{0}/2Q_{T}$, and $\Gamma_{0}=\omega_{0}/2Q_{0}$ for the resonator-*i*, load loop-*i*, source loop, and resonator-0, respectively. Similarly, unloaded quality factors are represented as $Q_{i}=\omega_{0}L_{i}/R_{i}$, $Q_{Ri}=\omega_{0}L_{Ri}/R_{Ri}$, $Q_{T}=\omega_{0}L_{T}/R_{T}$, and $Q_{0}=\omega_{0}L_{0}/R_{0}$, where $\omega_{0}=2\pi f_{0}$ is the angular resonant frequency. The parameters $U_{T0}$, $U_{0i}$, and $U_{Ri}$ govern the power transfer from the source loop to the transmitting resonator-0, from resonator-0 to resonator-*i*, and from resonator-*i* to load loop-*i*, respectively. When $U_{T0}$ is large, for example, most of the input power from the source reaches the resonator-0.

Using (3), the figures of merit can be represented by the product of the coupling coefficient and quality factors as

(4) $$\sqrt{U_{Ri}}=\frac{\kappa_{Ri}}{\sqrt{\Gamma_{i}\Gamma_{Ri}}}=k_{Ri}\sqrt{Q_{i}Q_{Ri}},\;\sqrt{U_{T0}}=\frac{\kappa_{T0}}{\sqrt{\Gamma_{T}\Gamma_{0}}}=k_{T0}\sqrt{Q_{T}Q_{0}},\;\sqrt{U_{0i}}=\frac{\kappa_{0i}}{\sqrt{\Gamma_{0}\Gamma_{i}}}=k_{0i}\sqrt{Q_{0}Q_{i}}.$$

In a well-designed WPT system, these parameters can be much greater than one for efficient power delivery. A small coupling coefficient at a large distance can be overcome by using a high-quality factor [3].

### 2.2. Analysis of Power Transfer and Division

**Figure 3:**
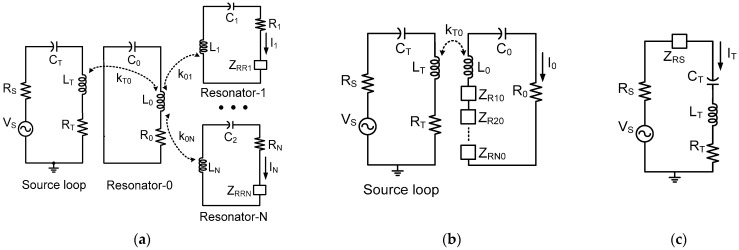
Simplified equivalent circuit of the resonator-coupled WPT system using (**a**) $Z_{RRi}$, (**b**) $Z_{Ri0}$, and (**c**) $Z_{RS}$.

Figure 2 can be transformed into Figure 3a using $Z_{RRi}$, which is the reflected impedance from load loop-i to receiving resonator-i, as

(5) $$Z_{RRi}=\frac{{\left(\omega_{0}M_{Ri}\right)}^{2}}{R_{Li}+R_{Ri}}=R_{i}k_{Ri}^{2}\frac{\omega_{0}L_{i}}{R_{i}}\frac{\omega_{0}L_{Ri}}{R_{Ri}}\frac{R_{Ri}}{R_{Li}+R_{Ri}}$$

where $M_{Ri}=k_{Ri}\sqrt{L_{Ri}L_{i}}$ is the mutual inductance between load loop-i and resonator-i. The result (5) can be further simplified as

(6) $$Z_{RRi}=R_{i}\frac{1}{\left(R_{Li}/R_{Ri}+1\right)}\frac{\omega_{0}^{2}k_{Ri}^{2}/4}{\Gamma_{i}\Gamma_{Ri}}\;=R_{i}\frac{1}{x_{i}+1}\frac{\kappa_{Ri}^{2}}{\Gamma_{i}\Gamma_{Ri}}=\frac{R_{i}}{x_{i}+1}U_{Ri}$$

where $x_{i}={(R}_{Li}/R_{Ri})$ is the normalized external loading, representing how much power is delivered to each load.

Figure 3a can be simplified to Figure 3b using the reflected impedance $Z_{Ri0}$, which is the impedance seen from the transmitting resonator-0 to the receiving resonator-i, as

(7) $$\begin{array}{ll} Z_{Ri0} & =\frac{{\left(\omega_{0}M_{0i}\right)}^{2}}{R_{i}+Z_{RRi}}=R_{0}k_{0i}^{2}\frac{\omega_{0}L_{0}}{R_{0}}\frac{\omega_{0}L_{i}}{R_{i}\left[1+U_{Ri}/\left(x_{i}+1\right)\right]}=R_{0}\frac{1}{\left[1+U_{Ri}/\left(x_{i}+1\right)\right]}\frac{\omega_{0}^{2}k_{0i}^{2}/4}{\Gamma_{0}\Gamma_{i}}\\ & =\frac{R_{0}}{1+U_{Ri}/\left(x_{i}+1\right)}U_{0i} \end{array}$$

where $M_{0i}=k_{0i}\sqrt{L_{0}L_{i}}$ is the mutual inductance between resonator-i and resonator-0.

Figure 3b can be further simplified to Figure 3c using the reflected impedance $Z_{RS}$, which is the impedance seen from the source loop to the resonator-0, as

(8) $$Z_{RS}={\left(\omega_{0}M_{T0}\right)}^{2}/\left(R_{0}+\sum_{i=1}^{N}Z_{Ri0}\right)$$

where $M_{T0}=k_{T0}\sqrt{L_{T}L_{0}}$ is the mutual inductance between the source loop and transmitting resonator-0. Using (7) in (8), $Z_{RS}$ can be rearranged as

(9) $$Z_{RS}=R_{T}k_{T0}^{2}\frac{\omega_{0}L_{T}}{R_{T}}\frac{\left(\omega_{0}L_{0}/R_{0}\right)}{1+\sum_{i=1}^{N}\frac{U_{0i}}{\left(1+U_{Ri}/\left(x_{i}+1\right)\right)}}\;=R_{T}\frac{U_{T0}}{1+\sum_{i=1}^{N}\frac{U_{0i}}{\left(1+U_{Ri}/\left(x_{i}+1\right)\right)}}.$$

Under resonance, the current $I_{T}$ in the source loop of Figure 3c can be expressed as

(10) $$I_{T}=\frac{V_{S}}{R_{S}+R_{T}+Z_{RS}}.$$

Using (10), the power $P_{0}$ transferred to resonator-0 can be expressed as

(11) P0=IT2Re(ZRS)=I02ReR0+∑i=1NZRi0.

Using (11), the current $I_{0}$ flowing in resonator-0 can be written as

(12) $$I_{0}=-j\frac{\sqrt{U_{T0}(R_{T}/R_{0})}}{1+\sum U_{N}}I_{T}$$

where $\sum U_{N}$ represents the overall loading of *N* receivers as

$$\sum U_{N}=\sum_{i=1}^{N}\frac{U_{0i}}{1+U_{Ri}/\left(x_{i}+1\right)}.$$

All the symbols used in this paper are listed in the Nomenclature.

Similarly, the power $P_{i}$ delivered to resonator-i can be expressed as

(13) Pi=I02Re(ZRi0)=Ii2ReRi+ZRRi

where the current $I_{i}$ flowing in the receiving resonator-*i*, which depends on multiple parameters, can be written as

(14) $$I_{i}=-j\frac{\sqrt{U_{0i}(R_{0}/R_{i})}}{1+U_{Ri}/\left(x_{i}+1\right)}I_{0}=-\frac{\sqrt{U_{T0}U_{0i}(R_{T}/R_{i})}}{\left[1+U_{Ri}/\left(x_{i}+1\right)\right]\left[1+\sum U_{N}\right]}I_{T}.$$

The power $P_{Loop,i}$ transferred to load loop-i can be obtained using

(15) PLoop,i=Ii2Re(ZRRi)=ILi2ReRRi+RLi.

Then, the current $I_{Li}$ in the load loop-i can be expressed as

(16) $$\left|I_{Li}\right|=\sqrt{\frac{U_{Ri}R_{i}/\left(x_{i}+1\right)}{\left(R_{Li}+R_{Ri}\right)}}\left|I_{i}\right|.$$

Using (14), the above result can be rearranged as

(17) $$\left|I_{Li}\right|=\frac{\sqrt{U_{T0}U_{0i}U_{Ri}R_{T}/\left(R_{Li}+R_{Ri}\right)\left(x_{i}+1\right)}}{\left\{1+U_{Ri}/\left(x_{i}+1\right)\right\}\left\{1+\sum U_{N}\right\}}\left|I_{T}\right|.$$

The ratio $r_{mn}$ of output power $P_{Ln}$ and $P_{Lm}$ for the load $R_{Ln}$ and $R_{Lm}$ is defined as

(18) $$r_{nm}=\frac{P_{Ln}}{P_{Lm}}=\frac{{\left|I_{Ln}\right|}^{2}}{{\left|I_{Lm}\right|}^{2}}\frac{R_{Ln}}{R_{Lm}}.$$

Using (17) in (18), we obtain

(19) $$r_{nm}=\frac{U_{0n}U_{Rn}x_{n}{\left(U_{Rm}+x_{m}+1\right)}^{2}}{U_{0m}U_{Rm}x_{m}{\left(U_{Rn}+x_{n}+1\right)}^{2}}.$$

If the receivers are identical ($x_{m}$ = $x_{n}$ and $U_{Rm}$ = $U_{Rn}$), the result (19) can be simplified as

(20) $$r_{nm}=\frac{U_{0n}}{U_{0m}}≅{\left(\frac{k_{0n}}{k_{0m}}\right)}^{2}$$

which is determined by the square of the coupling coefficients. The results indicate that $r_{mn}$ can be controlled by adjusting the distance ($d_{Ri}$) and/or position ($d_{0i}$ and $\rho_{0i}$) of the receivers.

To find the relation between $U_{Rm}$ and $U_{Rn}$, we solve (19) for the variable $U_{Rn}$ as

(21) $$U_{Rn}^{2}+\left\{2\left(x_{n}+1\right)-\frac{U_{0n}x_{n}{\left(U_{Rm}+x_{m}+1\right)}^{2}}{r_{nm}U_{0m}U_{Rm}x_{m}}\right\}U_{Rn}+{\left(x_{n}+1\right)}^{2}=0.$$

The solution of (21) can be expressed as

(22) $$U_{Rn}=\frac{1}{2}\left\{\frac{U_{0n}x_{n}{\left(U_{Rm}+x_{m}+1\right)}^{2}}{r_{nm}U_{0m}U_{Rm}x_{m}}-2\left(x_{n}+1\right)\pm \sqrt{\Delta }\right\}$$

where

$$\Delta =\frac{U_{0n}x_{n}{\left(U_{Rm}+x_{m}+1\right)}^{2}}{r_{nm}U_{0m}U_{Rm}x_{m}}\left\{\frac{U_{0n}x_{n}{\left(U_{Rm}+x_{m}+1\right)}^{2}}{r_{nm}U_{0m}U_{Rm}x_{m}}-4\left(x_{n}+1\right)\right\}.$$

The condition (Δ> 0) leads to the following relation:

(23) $$\frac{U_{0n}x_{n}{\left(U_{Rm}+x_{m}+1\right)}^{2}}{r_{nm}U_{0m}U_{Rm}x_{m}}-4\left(x_{n}+1\right)>0.$$

Solving (23) with variable $x_{n}$, we obtain

(24) $$x_{n}>{\left[\frac{U_{0n}}{r_{nm}}\frac{{\left(U_{Rm}+x_{m}+1\right)}^{2}}{4U_{0m}U_{Rm}x_{m}}-1\right]}^{-1}.$$

When the condition (24) is satisfied, the above result shows that $r_{nm}$ can be obtained by choosing $U_{Rn}$ given by (22). In practice, $\;U_{Rn}$ can be adjusted by changing the coupling $k_{Ri}$ between load loop-*n* and receiving resonator-*n*.

### 2.3. Analysis of Efficiency

The system efficiency $\eta_{E}$ can be calculated using the output load power $P_{Li}$, input power $P_{IN}$, and the loss $P_{LOSS}$ as

(25) $$\eta_{E}=\frac{\sum_{i=1}^{N}P_{Li}}{P_{IN}+P_{LOSS}+\sum_{n=1}^{N}P_{Li}}=\frac{\sum_{i=1}^{N}{\left|I_{Li}\right|}^{2}R_{Li}}{{\left|I_{T}\right|}^{2}\left(R_{S}+R_{T}\right)+{\left|I_{0}\right|}^{2}R_{0}+\sum_{i=1}^{N}{\left|I_{i}\right|}^{2}R_{i}+\sum_{i=1}^{N}{\left|I_{Li}\right|}^{2}\left(R_{Li}+R_{Ri}\right)}.$$

Using (12), (14), and (17), $\eta_{E}$ can be rearranged as

(26) $$\eta_{E}=\frac{\frac{U_{T0}}{{\left\{1+\sum U_{N}\right\}}^{2}}\left\{\sum_{i=1}^{N}\frac{U_{0i}U_{Ri}}{{\left\{1+U_{Ri}/\left(x_{i}+1\right)\right\}}^{2}}\frac{x_{i}}{{\left(x_{i}+1\right)}^{2}}\right\}}{R_{S}/R_{T}+1+\frac{U_{T0}}{{\left\{1+\sum U_{N}\right\}}^{2}}\left\{1+\sum U_{N}+\sum_{i=1}^{N}\frac{U_{0i}U_{Ri}}{{\left\{1+U_{Ri}/\left(x_{i}+1\right)\right\}}^{2}}\frac{1}{x_{i}+1}\right\}}.$$

The above result can be further simplified as

(27) $$\eta_{E}=\frac{\sum_{i=1}^{N}U_{Ri}U_{0i}x_{i}}{\frac{\left(R_{S}/R_{T}+1\right){\left[\left(1+x_{i}\right)+U_{Ri}\right]}^{2}{\left(1+\sum U_{N}\right)}^{2}}{U_{T0}}+{\left[\left(1+x_{i}\right)+U_{Ri}\right]}^{2}+\sum_{i=1}^{N}U_{0i}\left(1+x_{i}\right)\left(\left(1+x_{i}\right)+U_{Ri}\right)}.$$

In high-power applications, $\eta_{E}$ is usually considered, which includes the loss of the system. Under the impedance matching condition, the maximum power is delivered to the load. Half of the power is dissipated in the power source, resulting in $\eta_{E}$ = 50% when $P_{LOSS}$ is neglected. When ($R_{S}$ + $R_{T}$) is reduced, the power dissipated in the power source decreases, and most of the power is delivered to the load, resulting in $\eta_{E}$ > 50% [33]. In low-power, midrange WPT applications, the loss of the power source is not of primary concern. And, we can consider power transfer efficiency $\eta_{T}$, which can be easily measured using a network analyzer and standard calibration technique. When multiple receivers are used, $\eta_{Ti}$ of each receiver can be defined using the ratio of $P_{Li}$ and $P_{IN}$ (*i* = 1 – *N*). The overall $\eta_{T}$ is the sum of the transmission efficiency of each receiver as follows:

(28) $$\eta_{T}=\sum_{i=1}^{N}\eta_{T,i}=\sum_{i=1}^{N}\frac{P_{Li}}{P_{IN}}=\frac{1}{(V_{S}^{2}/4R_{S})}\sum_{i=1}^{N}{\left|I_{Li}\right|}^{2}R_{Li}.$$

Using (17) in (28), we obtain

(29) $$\eta_{T}=\frac{1}{(V_{S}^{2}/4R_{S})}\sum_{i=1}^{N}\frac{U_{T0}U_{0i}U_{Ri}R_{T}/\left(R_{Li}+R_{Ri}\right)\left(x_{i}+1\right)}{{\left\{1+U_{Ri}/\left(x_{i}+1\right)\right\}}^{2}{\left\{1+\sum U_{N}\right\}}^{2}}I_{T}^{2}R_{Li}.$$

Using (9) and (10), the relation between $V_{S}$ and $I_{T}$ can be expressed as

(30) $$\frac{V_{S}}{I_{T}}=\left(R_{S}+R_{T}\right)+\frac{R_{T}U_{T0}}{1+\sum U_{N}}$$

Using (30) in (29), $\eta_{T}$ can be rearranged as

(31) $$\eta_{T}=\frac{4U_{T0}(R_{S}/R_{T})}{{\left[(R_{S}/R_{T}+1)(1+\sum U_{N})+U_{T0}\right]}^{2}}\sum_{i=1}^{N}\frac{U_{0i}U_{Ri}x_{i}}{{\left[\left(x_{i}+1\right)+U_{Ri}\right]}^{2}}.$$

To find the condition for maximizing ${\;\eta }_{T}$ when other parameters are given, we take the derivative of $\eta_{T}$ with respect to $U_{T0}$ and set it to zero, which results in the optimum value $U_{T0,opt}$ as follows:

(32) $$U_{T0,opt}=\left(1+\frac{R_{S}}{R_{T}}\right)\left[1+\sum_{i=1}^{N}\frac{U_{0i}}{\left(1+U_{Ri}/\left(x_{i}+1\right)\right)}\right].$$

**Figure 4:**
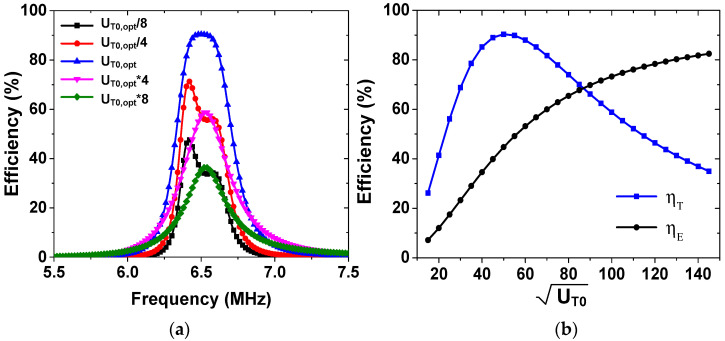
(**a**) Simulated power transfer efficiencies as a function of frequency for different values of $U_{T0}$. (**b**) Calculated efficiencies as a function of $\sqrt{U_{T0}}$.

Figure 4a shows simulated $\eta_{T}$ as a function of frequency for different values of $U_{T0}$. The Advanced Design System is used for circuit simulations, with the parameters shown in Table 1. All coils and resonators are assumed to have the same frequency ($f_{0}$ = 6.5 MHz). The peak efficiency of 90.4% at 6.5 MHz is achieved when $U_{T0}$ = $U_{T0,opt}$ is used. When $U_{T0}$ deviates from $U_{T0,opt}$, $\eta_{T}$ decreases, and the frequency where the peak efficiency occurs shifts to a lower frequency. Figure 4b shows the calculated $\eta_{T}$ and $\eta_{E}$ as a function of $\sqrt{U_{T0}}$. $\eta_{E}$ increases up to 82.5% at $\sqrt{U_{T0}}$ = 145. A peak $\eta_{T}$ value of 90.4% is achieved at $\sqrt{U_{T0}}$ = 50, with a corresponding $\eta_{E}$ value of 44.7%.

## 3. Experimental Results

For the experiment, a WPT system consisting of one transmitter and four receivers is fabricated. The transmitter and each receiver include a pair of copper coils: one loop coil and one resonator. The transmitting resonator-0 has a spiral shape with a turn of 5, a pitch of 1.5 cm, and an inner radius of 20 cm. The receiving resonator-*i* has a smaller spiral with a turn of 10, a pitch of 0.5 cm, and an inner radius of 4 cm. The source and load loops are fabricated using a single-turn coil with a radius of 20 cm and 6 cm, respectively. The diameter of the copper wire is 5 mm and 2.5 mm for transmitting and receiving coils, respectively. Because the inductance of the resonator is quite large (~nH), the resonant frequency is sensitive to a slight change in capacitance; a fixed capacitor is added in series with coils to set $f_{0}$ close to 6.5 MHz. The measured coil parameters are shown in Table 2.

**Figure 5:**
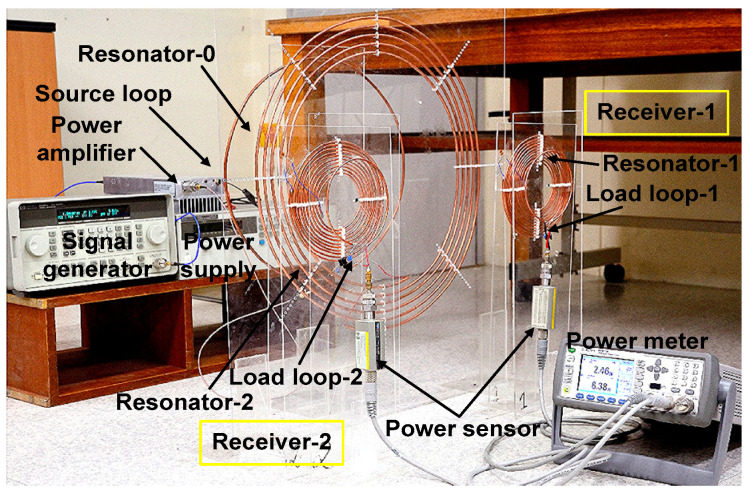
Experimental setup of the resonator-coupled WPT system for powering two receivers.

Figure 5 shows the experiment setup when two receivers are used. A signal generator and an RF power amplifier connected to the source loop provide input power $P_{IN}$. A power meter with two power sensors is used to measure load power. In the first case, we set the power ratio $r_{21}$ = 1.5 between two receivers. The vertical distance ($d_{0i}$) and lateral displacement ($\rho_{0i}$) of the two receivers are set as $d_{01}=d_{02}$= 15 cm and $\rho_{01}=\rho_{02}=$ 23 cm. The separation of 46 cm between the two receivers results in a coupling coefficient of 1.6 × 10^−4^, which is sufficiently small to be negligible. Using the measured *Q*-factor and coupling coefficient, we obtain $U_{R1}$ = 12,555. Using (22), the calculated value is $U_{R2}$ = 8313. Using this value in (32), $U_{T0,opt}$ = 2612 is obtained.

During the experiment, the locations of resonator-1 ($U_{01}$) and load loop-1 ($U_{R1}$) for the receiver-*1* are fixed ($d_{R1}$ = 3.8 cm), and the two parameters of $d_{R2}$ and $d_{T0}$ are adjusted. Then, load loop-2 ($U_{R2}$) is moved step by step until $r_{21}$ = 1.5 is achieved. This procedure results in $d_{R2}≅$ 2 cm. Previous work shows that impedance matching can be achieved by varying the coupling coefficient (or distance) between the source (load) loop and the internal resonator [4]. In this paper, we apply the variable coupling method for asymmetric multiple receivers. Using this technique, $d_{T0}$ is moved slowly until $\eta_{T}$ reaches a peak value, resulting in the optimum distance $d_{T0,opt}$ = 12 cm corresponding to $U_{T0,opt}$.

**Figure 6:**
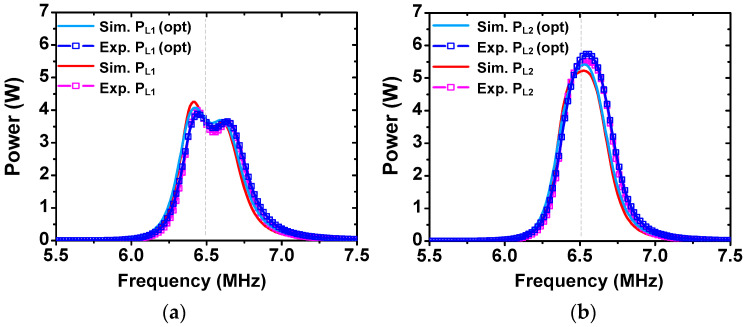
Measured and simulated load power of (**a**) receiver-1 and (**b**) receiver-2 for the power ratio of 1.5.

Figure 6 shows the measured and simulated $P_{L1}$ and $P_{L2}$ of the two receivers when $P_{IN}$ = 10 W. Before tuning $d_{T0}$, the simulated power ratio is $P_{L2}/P_{L1}\;$= 5.21 W/3.59 W = 1.45. The measured ratio is $P_{L2}/P_{L1}\;$= 5.44 W/3.55 W = 1.53 at 6.5 MHz, which is close to target $r_{21}$ values. After tuning optimization, it shows a simulated $P_{L2}/P_{L1}\;$= 5.35 W/3.69 W= 1.45 and measured $P_{L2}/P_{L1}\;$= 5.55 W/3.62 W= 1.53. The result shows that $r_{21}$ is close to the target value of 1.5 both before and after optimization. After tuning $d_{T0}$ for impedance matching, the measured $P_{L1}$ and $P_{L2}$ are slightly improved by 0.07 W and 0.11 W, respectively; the initial position of the source loop ($d_{T0}=15$ cm) is close to $d_{T0,opt}=12$ cm. After tuning, $\eta_{T}$ improves from 89.9% to 91.7%. The parameters of the transmitter and receiver used for the experiment are listed in Table 3 and Table 4, respectively.

**Figure 7:**
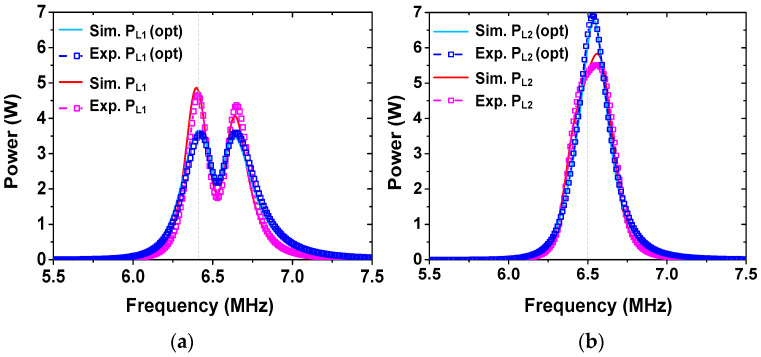
Measured and simulated load power of (**a**) receiver-1 and (**b**) receiver-2 for the power ratio of 2.5.

In the second case, we set a new power ratio $r_{21}$ = 2.5 between the two receivers. The same vertical distance ($d_{0i}$) and lateral displacement ($\rho_{0i}$) are used. Calculated values of $U_{R2}=$ 4770 and $U_{T0,opt}=$ 3675 are obtained using (22) and (32), respectively. During the experiment, we perform similar steps to the first case to obtain $d_{R2}≅3$ cm for the target $r_{21}$ value. Figure 7 shows the measured and simulated $P_{L1}$ and $P_{L2}$. Before tuning $d_{T0}$, the result shows the simulated $P_{L2}/P_{L1}$ = 5.29 W/2.19 W = 2.42 and measured $P_{L2}/P_{L1}$ = 5.3 W/2.09 W = 2.54 at 6.5 MHz, which are close to target $r_{21}$ values. After tuning $d_{T0}$ for impedance matching, we obtain $d_{T0}≅$ 10 cm. It shows the simulated $P_{L2}/P_{L1}\;$= 6.08 W/2.52 W = 2.41 and measured $P_{L2}/P_{L1}$ = 6.38 W/2.48 W = 2.57. The measured $P_{L1}$ and $P_{L2}$ are improved by 0.39 W and 1.08 W, respectively, and $r_{21}$ is still close to the target value. After tuning, $\eta_{T}$ is improved from 73.9% to 88.6%. The results show that the proposed WPT system can achieve power division with a relatively high efficiency. The difference between the simulated and measured output power may be attributed to the slightly different resonant frequencies and *Q*-factors of the two receivers, inter-resonator coupling, and frequency-dependent loss not considered in the simulation model.

**Figure 8:**
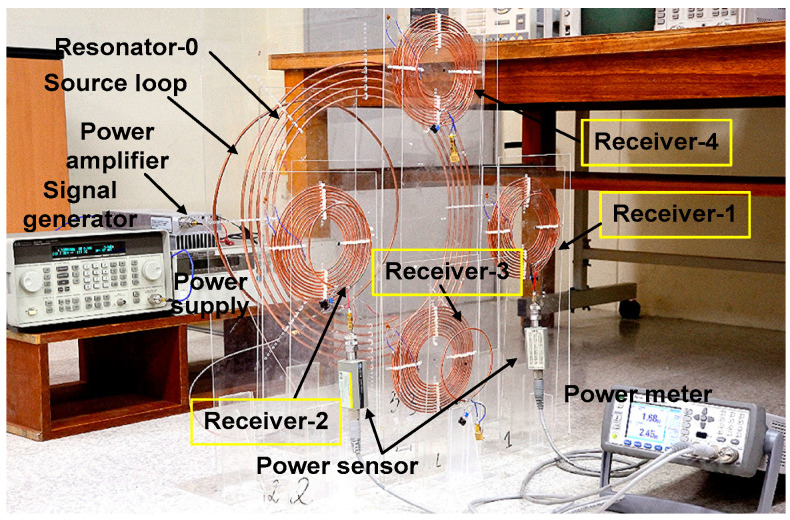
Experimental setup of the resonator-coupled WPT system for powering four receivers.

Figure 8 shows the experiment setup when four receivers are used. Load loops not connected to power sensors are terminated with 50 Ω. Experiments are performed for target division ratios of $r_{21}=$ 1.5, $r_{31}=$ 2.0, and $r_{41}=$ 0.75. The vertical distance of the four receivers is set as $d_{01}=d_{02}\;$= $d_{03}\;$= $d_{04}\;$= 15 cm. The lateral displacement of the receivers is set as $\rho_{01}=\rho_{02}=$ 23 cm, $\rho_{03}=$ 24 cm, and $\rho_{04}=$ 25 cm. Under these conditions, the simulated coupling coefficient is 1.6 × 10^−4^ between nonadjacent receivers and 1.7 × 10^−3^ between adjacent receivers. These values are significantly smaller than the primary coupling between the transmitter and receiver. Using (22), we obtain $U_{R2}=$ 8294, $U_{R3}=$ 3164, $U_{R4}=$ 9965. $U_{T0,opt}=$ 5081 is obtained using (31). During the experiment, similar steps are performed for two receivers. We experimentally obtain $d_{R2}≅$ 2 cm, $d_{R3}≅$ 4 cm, and $d_{R4}≅$ 1.5 cm.

**Figure 9:**
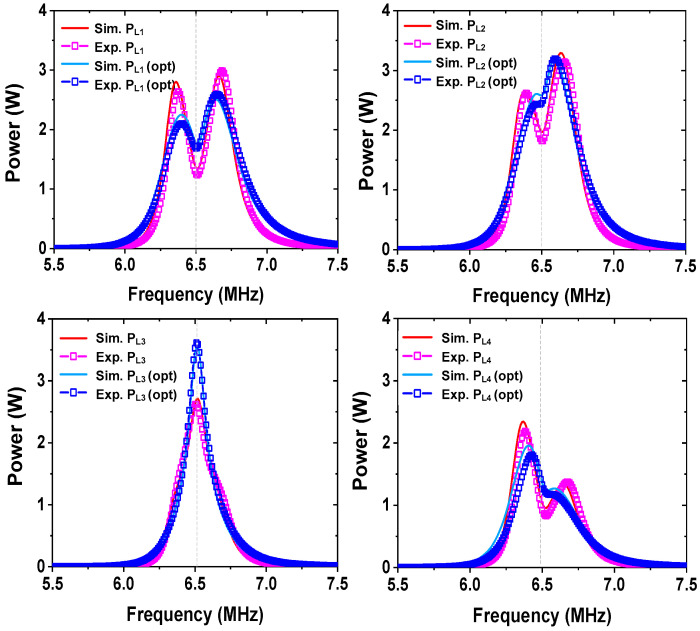
Measured and simulated load power of the four receivers.

Figure 9 shows the measured and simulated load power of four receivers. Before tuning $d_{T0}$, the simulated values are $P_{L2}/P_{L1}$ = 1.97 W/1.36 W = 1.45, $P_{L3}/P_{L1}$ = 2.63 W/1.36 W = 1.93, and $P_{L4}/P_{L1}$ = 1.03 W/1.36 W = 0.76 at 6.5 MHz. The measured values are $P_{L2}/P_{L1}$ = 1.82 W/1.25 W = 1.46, $P_{L3}/P_{L1}$ = 2.62 W/1.25 W = 2.1, and $P_{L4}/P_{L1}$ = 0.92 W/1.25 W = 0.74. The division ratios are close to the target value. The small difference can be attributed to slightly different resonant frequencies of coils and non-zero cross-couplings. After achieving the power ratios, $d_{T0}$ is tuned to achieve the peak $\eta_{T}$ value. The experimentally determined value for peak $\eta_{T}$ is $d_{T0,opt}≅\;$7 cm. The measured division ratios are $P_{L2}/P_{L1}$ = 2.45/1.69 = 1.45, $P_{L3}/P_{L1}$ = 3.53/1.69 = 2.09, and $P_{L4}/P_{L1}$ = 1.33/1.69 = 0.79. The power ratios after tuning $d_{T0}$ are maintained as relatively constant; the power delivered to loads is improved by 0.44 W for $P_{L1}$, 0.63 W for $P_{L2}$, 0.91 W for $P_{L3}$, and 0.41 W for $P_{L4}$. After tuning, $\eta_{T}$ is significantly improved from 66.1% to 90%. The results show that the proposed WPT system can achieve the target power division for the four receivers with a relatively high efficiency. The parameters of the four receivers used for the experiment are listed in Table 5.

**Figure 10:**
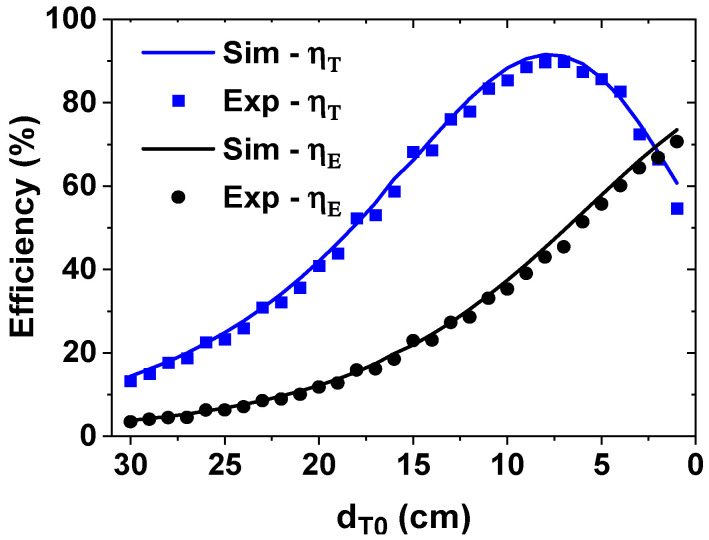
Measured and calculated efficiencies as a function of $d_{T0}$.

Figure 10 shows the simulated and calculated efficiencies as a function of $d_{T0}$. The result shows that peak $\eta_{T}$ = 89.9% is achieved at $d_{T0,opt}$ = 7 cm, with a corresponding $\eta_{E}$ value of 45.4%. $\eta_{E}$ increases with decreasing $d_{T0}$, reaching 70.7% at $d_{T0}$ = 1 cm. The measured results show a relatively good agreement with the simulated data. Among the variable parameters ($d_{0i}$, $\rho_{0i},\;d_{Ri}$, and $d_{T0}$) considered in this work, $d_{Ri}$ and $d_{T0}$ are adjusted while $d_{0i}$ and $\rho_{0i}$ are fixed during the experiment to simplify the experimental procedure. Therefore, the two parameters ($d_{0i}$ and $\rho_{0i}$) can also affect the efficiency when they are varied. To determine the four variables for the demanded power ratio, in this prototype, power sensors are used. In practical applications demanding automated control, measured power values corresponding to the four variables can be stored in the lookup table (see Figure 1). When the desired power ratio information is input to the position controller, the position information is retrieved from the table, and positioning control can be performed using the parameter sets ($d_{0i}$, $\rho_{0i},\;d_{Ri}$, and $d_{T0}$).

To investigate the sensitivity of distance around the optimal value, we can consider the case when $d_{T0}$ deviates from $d_{T0,opt}$. When $d_{T0}$ is reduced by 1 cm ($d_{T0}$ = 6 cm), $\eta_{E}$ increases to 51.4% while $\eta_{T}$ decreases to 87.4%. When $\eta_{E}$ increases above 50%, the delivered power to the load decreases [33]. When $d_{T0}$ is increased by 1 cm ($d_{T0}$ = 8 cm), $\eta_{E}$ decreases to 42.9% and $\eta_{T}$ decreases to 89.7%. Because $\eta_{T}$ shows a peak value at the impedance matching condition, it reduces when $d_{T0}$ deviates from $d_{T0,opt}$. The results show that $\eta_{T}$ gradually decreases beyond the optimal distance. However, the drop-off is gradual because our system contains high-Q resonators that help to maintain a stable operation around the optimal position. Table 6 shows the comparison with the previous works.

## 4. Conclusions

This paper investigates the asymmetric resonant coupling WPT system for powering multiple receivers. We propose a simple method for achieving the power ratio for each receiver using the equivalent circuit model and reflected impedance technique. The results are generalized for the system with an *N* number of multiple receivers. This work also demonstrates that the variable coupling method increases the power transfer efficiency under asymmetric multiple-receiver conditions. Unlike conventional approaches requiring complex impedance matching networks or additional circuit components, our method achieves the nonuniform powering of multiple receivers through spatial positioning and impedance tuning, allowing for flexible and scalable implementation. Experiments are performed for powering two receivers with different power ratios (1.5 and 2.5). Using straightforward experimental steps, the desired power ratio is achieved with a relatively high efficiency of up to 91.7%. Another experiment performed for powering four receivers with different power ratios (1.5, 2.0, and 0.75) shows an efficiency of up to 89.9%, which agrees well with the simulation result. Further work is needed to implement the position controller and lookup table for automated control. The proposed method can be applied to a system where a large transmitter delivers power to multiple small receivers, such as wearable IoT sensors and universal charging pads.

## Figures and Tables

**Table 1:** Parameters of transmitter and receivers used for simulation.

Transmitter	Receiver-1	Receiver-2
$R_{s}$ = 50 Ω	*R*_L1_ = 50 Ω	*R*_L2_ = 50 Ω
$\Gamma_{T}$ = 2.12 × $10^{5}$	$\Gamma_{1}$ = 2.26 × $10^{4}$	$\Gamma_{2}$ = 2.58 × $10^{4}$
$\Gamma_{0}$ = 3.15 × $10^{4}$ $k_{To}$ = 0.06–0.6 $U_{T0}$ = 225–22,468	$\Gamma_{R1}$ = 3.32 × $10^{5}$ $k_{01}$ = 0.0408 $k_{R1}$ = 0.475 $U_{01}$ = 976 $U_{R1}$ = 12,555 $x_{1}$ = 238.1	$\Gamma_{R2}$ = 2.89 × $10^{5}$ $k_{02}$ = 0.0517 $k_{R2}$ = 0.386 $U_{02}$ = 1372 $U_{R2}$ = 8313 $x_{2}$ = 277.8

**Table 2:** Measured coil parameters.

Transmitter	Inductance (nH)	Resistance (Ω)	Freq. (MHz)	Q-Factor
Source loop	1180	0.5	6.5	$Q_{T}=$96.3
Resonator-0	20,800	1.3	6.45	$Q_{0}=$648.1
Load loop-1	316	0.21	6.51	$Q_{R1}=$61.5
Resonator-1	15,540	0.7	6.49	$Q_{1}=$ 904.8
Load loop-2	310	0.18	6.51	$Q_{R2}=$70.4
Resonator-2	15,580	0.8	6.48	$Q_{2}=$ 792.5
Load loop-3	321	0.26	6.51	$Q_{R3}=$50.5
Resonator-3	15,500	0.76	6.52	$Q_{3}=$ 835.1
Load loop-4	319	0.23	6.48	$Q_{R4}=$56.4
Resonator-4	15,550	0.72	6.52	$Q_{4}=$884.3

**Table 3:** Parameters of the transmitter used for the experiment.

	${\;R}_{s}$	$\Gamma_{T}$	$\Gamma_{0}$	$d_{T0}$
Transmitter	50 Ω	2.12 × $10^{5}$	3.13 × $10^{4}$	1–30 cm

**Table 4:** Parameters of two receivers used for the experiment.

	$R_{Li}$	$\Gamma_{i}$	$\Gamma_{Ri}$	$k_{0i}$	$k_{Ri}$	$U_{0i}$	$U_{Ri}$	$x_{i}$
Receiver-1	50 Ω	2.25 × $10^{4}$	3.32 × $10^{5}$	0.03	0.475	528	12,555	238
Receiver-2	50 Ω	2.57 × $10^{4}$	2.91 × $10^{5}$	0.03	0.387	462	8313	278

**Table 5:** Parameters of four receivers used for the experiment.

	$R_{Li}$	$\Gamma_{i}$	$\Gamma_{Ri}$	$k_{0i}$	$k_{Ri}$	$U_{0i}$	$U_{Ri}$	$x_{i}$
Receiver-1	50 Ω	2.25 × $10^{4}$	3.32 × $10^{5}$	0.03	0.475	528	12,572	238
Receiver-2	50 Ω	2.57 × $10^{4}$	2.91 × $10^{5}$	0.03	0.386	462	8294	278
Receiver-3	50 Ω	2.45 × $10^{4}$	4.05 × $10^{5}$	0.024	0.274	312	3164	192
Receiver-4	50 Ω	2.32 × $10^{4}$	3.63 × $10^{5}$	0.023	0.447	303	9965	217

**Table 6:** Comparison with the previous works.

	[9]	[10]	[16]	[23]	[26]	[28]	This Work
WPT type	Resonant	Resonant	Resonant	Resonant	Resonant	Resonant	Resonant
No. of coils *	4	3	2	2	3	2	4
No. of receivers	1	1	1	4	3	3	4
No. of transmitters	1	2	1	1	1	1	1
Area ratio of Tx and Rx	28:1	1:1	1.86:1	29:1	8.4:1	3:1	9:1
Power division to multiple Rx	N	N	N	Y	Y	Y	Y
Unequal power division	N	N	N	N	N	Y	Y
Frequency (MHz)	13.56	0.15	1	13.56	6.7	1.28	6.5
Peak efficiency (%)	64	94.2	93.6	40.4	88.3	65	91.7

* For each pair of Tx and Rx.

## Data Availability

The datasets used and/or analysed during the current study available from the corresponding author on reasonable request.

## Nomenclature

The symbols used in this paper are listed below.

Symbol	Definition
$L_{T}$, $L_{0}$, $L_{i}$, and $L_{Ri}$	Inductance of the source loop, resonator-0, resonator-*i*, and load loop-*i*
$C_{T}$, $C_{0}$, $C_{i}$, and $C_{Ri}$	Capacitance of the source loop, resonator-0, resonator-*i*, and load loop-*i*
$R_{T}$, $R_{0}$, $R_{i}$, and $R_{Ri}$	Resistance of the source loop, resonator-0, resonator-*i*, and load loop-*i*
$R_{S}$ and $R_{Li}$	Source resistance and load resistance
$d_{T0}$, $d_{0i}$, and $d_{Ri}$	Vertical distance between the source loop and resonator-0, resonator-0 and resonator-*i*, and resonator-*i* and load loop-*i*
$\rho_{0i}$	Lateral displacement from the center of resonator-0
$k_{T0}$$,\;k_{Ri}$, and $k_{0i}$	Coupling coefficient of the transmitter, load, and receiver
$\kappa_{T0}$$,\;\kappa_{Ri}$, and $\kappa_{0i}$	Normalized coupling coefficient of the transmitter, load, and receiver
$U_{T0}$, $U_{Ri}$, and $U_{0i}$	Coupling factor of the transmitter, load, and receiver
$\Gamma_{T}$, $\Gamma_{0}$, $\Gamma_{i}$, and $\Gamma_{Ri}$	Loss rate of the source loop, resonator-0, resonator-*i*, and load loop-*i*
$Q_{T},\;Q_{0},\;Q_{i},\;\mathrm{and}\;Q_{Ri}$	Quality factor of the source loop, resonator-0, resonator-*i*, and load loop-*i*
$Z_{RRi}$, $Z_{Ri0}$, and $Z_{RS}$	Reflected impedance seen from the load loop-*i* to resonator-*i*, from resonator-*i* to resonator-0, and from resonator-0 to the source loop
$M_{T0}$, $M_{0i}$, and $M_{Ri}$	Mutual inductance between the source loop and resonator-0, resonator-0 and resonator-*i*, and resonator-*i* and load loop-*i*
$x_{i}$	$\mathrm{Normalized}\;\mathrm{external}\;\mathrm{loading}\;(=R_{Li}/R_{Ri})$
$I_{T}$, $I_{0}$, $I_{i}$, and $I_{Li}$	Current in the source loop, resonator-0, resonator-*i*, and load loop-*i*
$P_{0}$, $P_{i}$, and $P_{Loop,i}$	Power transferred to the resonator-0, resonator-*i*, and load loop-*i*
$P_{IN}$, $P_{LOSS}$, and $P_{Li}$	Input power, loss power, and output load power
$\Sigma U_{N}$	Overall loading of *N* receivers
$r_{mn}$	Power ratio between two receivers
$\eta_{E}$ and $\eta_{T}$	System efficiency and overall power transfer efficiency
$U_{T0,opt}$	Optimal coupling value for maximum power transfer efficiency
$d_{T0,opt}$	$\mathrm{Optimal}\;\mathrm{distance}\;\mathrm{corresponding}\;\mathrm{to}\;U_{T0,opt}$
